# Risk Factors Associated With Burden of Disease of Psoriasis From 1990 to 2019: Epidemiological Analysis

**DOI:** 10.2196/48749

**Published:** 2024-02-15

**Authors:** Vishnutheertha Kulkarni, David Liu, Vahram Gamsarian, Tjinder Grewal, Torunn Sivesind

**Affiliations:** 1 University of Queensland Woolongabba Australia; 2 University of Toledo Toledo, OH United States; 3 University of Michigan Medical School Ann Arbor, MI United States; 4 Department of Dermatology University of Colorado Anschutz Medical Campus Aurora, CO United States

**Keywords:** psoriasis, dermatology, gross domestic product, epidemiology, sociodemographic index, Global Burden of Disease, obesity, burden, skin, epidemiological, sociodemographic, chronic, noncommunicable, autoimmune, inflammation, inflammatory

## Introduction

Psoriasis is a chronic inflammatory skin condition characterized by red, itchy, scaly patches that affects approximately 2% of the global population and has a significant effect on the patient’s quality of life [1]. Exploring epidemiological trends and relevant risk factors for psoriasis is vital to effectively reduce the global burden of the disease by directing efforts toward countries with the highest prevalence. This study aims to characterize trends in global rates of psoriasis and their associations with relevant risk factors.

## Methods

We obtained global psoriasis data from the University of Washington Institute for Health Metrics and Evaluation Global Burden of Disease (GBD) Database and sorted it by age-standardized incidence, prevalence, and years lost to disability (YLD) rates per 100,000 people from 1990 to 2019 [2]. We further filtered these metrics by the four world regions (Asia, Africa, America, and Europe), sociodemographic index (SDI) quintiles, and the 204 countries/territories listed in the GBD database. Country-level indicator data was extracted from the World Health Organization Global Health Observatory database for possible associations with psoriasis [3]. Linear regression analyses were conducted between risk factors and incidence, prevalence, and YLD rates of psoriasis.

### Ethical Considerations

This paper was conducted using publicly available databases. Therefore, no ethics approval was required.

## Results

**Figure 1:**
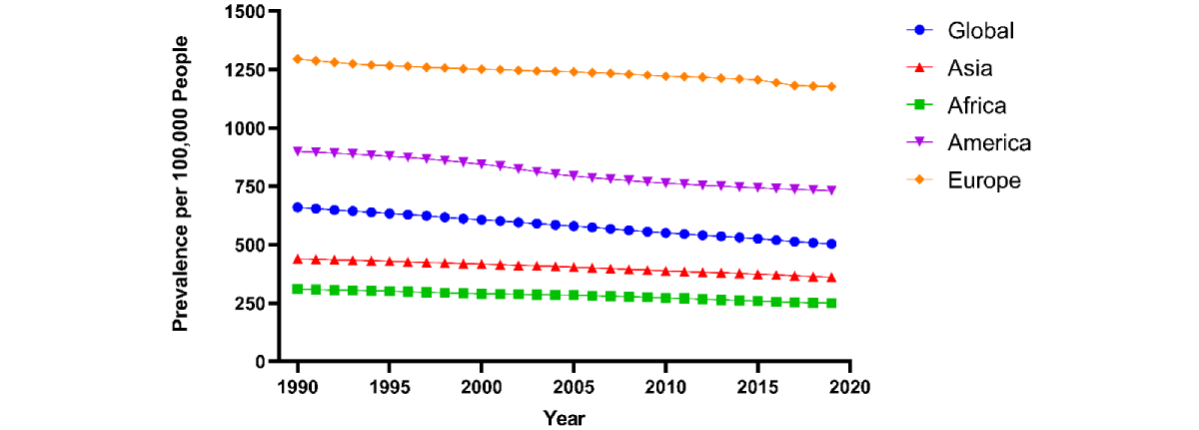
Global age-standardized prevalence rates of psoriasis per 100,000 people by Global Burden of Disease World Region.

The global age-standardized prevalence rate of psoriasis per 100,000 people in 1990 was 660 (95% CI 637-681). It decreased to 504 (95% CI 487-519) in 2019. Across the world regions, psoriasis prevalence, incidence, and YLD were highest in Europe and lowest in Africa (Figure 1). Psoriasis prevalence rates were higher in the highest quintile of SDI (1990: 1256; 2019: 1073) than in the lowest quintile of SDI (1990: 338, 2019: 301) from 1990 to 2019. Similar trends were found for incidence and YLD rates.

Psoriasis incidence rates were positively associated with overweight prevalence (*R*^2^=0.36), mean cholesterol (*R*^2^=0.21), mental hospital admissions (*R*^2^=0.25), medical doctors (*R*^2^=0.50), and psychiatrists in the mental health sector (*R*^2^=0.58) while being negatively associated with air pollution mortality rates (*R*^2^=0.40; Table 1). Similar trends were noted for risk factor associations with psoriasis prevalence and YLD rates (*P*<.001).

**Table 1:** Linear regression analyses of risk factors associated with incidence, prevalence, and years lost to disability (YLD) rates of psoriasis.

Risk factors and Y value		Association		Countries, n			*R* ^2^			*P* value	
**Overweight prevalence (BMI ≥25, age-standardized estimate; %)**					182						
	Incidence of psoriasis	Positive				0.26			<.001		
	Prevalence of psoriasis	Positive				0.22			<.001		
	YLDs of psoriasis	Positive				0.19			<.001		
**Mean total cholesterol (age-standardized estimate)**					184						
	Incidence of psoriasis	Positive				0.31			<.001		
	Prevalence of psoriasis	Positive				0.26			<.001		
	YLDs of psoriasis	Positive				0.23			<.001		
**Mortality rate attributed to household and ambient air pollution per 100,000 population (age-standardized)**					182						
	Incidence of psoriasis	Negative				0.40			<.001		
	Prevalence of psoriasis	Negative				0.35			<.001		
	YLDs of psoriasis	Negative				N/A^a^			<.001		
**Mental hospital admissions per 100,000 population**					98						
	Incidence of psoriasis	Positive				0.25			<.001		
	Prevalence of psoriasis	Positive				0.21			<.001		
	YLDs of psoriasis	Positive				0.17			<.001		
**Medical doctors per 100,000 population**					184						
	Incidence of psoriasis	Positive				0.50			<.001		
	Prevalence of psoriasis	Positive				0.44			<.001		
	YLDs of psoriasis	Positive				0.41			<.001		
**Psychiatrists working in mental health sector (per 100,000 population)**					102						
	Incidence of psoriasis	Positive				0.58			<.001		
	Prevalence of psoriasis	Positive				0.56			<.001		
	YLDs of psoriasis	Positive				0.53			<.001		

^a^N/A: not applicable.

## Discussion

There are a few reasons why global psoriasis prevalence consistently decreased since 1990. Psoriasis may go into remission, decreasing the duration of the disease and ultimately its prevalence, especially in older individuals. Additionally, comorbidities and adverse health behaviors may lead to increased mortality rates among individuals with psoriasis, resulting in decreased prevalence rates [4]. However, a significant global disease burden remains. Europe has the highest incidence, while Africa has the lowest. These findings were consistent with a prior study on the epidemiology of psoriasis [5]. Factors that were characteristic of wealthier countries such as high SDI, high overweight prevalence, higher mean cholesterol, and lower air pollution mortality rates were found to be associated with higher psoriasis incidence, prevalence, and YLD. Despite greater access to medical resources, high psoriasis prevalence in the highest SDI countries remains. Strong positive associations between psoriasis rates and medical doctors per 100,000 population and psychiatrists per 100,000 population further highlight this trend, underscoring the burden of psoriasis in areas more densely populated with medical professionals. Additionally, psoriasis rates are associated with mental hospital indications, indicating possible psychiatric comorbidities among patients with psoriasis. Solutions must be tailored to more complex causes of psoriasis, such as the gut-brain-skin axis’ role in skin disorders, smoking exposure, alcohol intake, specific medications, and even genetic causes [6].

Limitations of this study include underreporting in some sub-Saharan regions and potentially inaccurate modeling algorithms by the GBD website. Additionally, there may potentially be an ecological fallacy as the populations analyzed in this study may not be representative of the individual members. This study provides a unique and recent perspective on the epidemiological trends of psoriasis. To effectively reduce the burden of psoriasis in these countries, more research on the complex environmental and genetic risk factors of psoriasis should be conducted.

## Abbreviations

GBD: Global Burden of Disease
SDI: sociodemographic index
YLD: years lost to disability
